# Cryptococcal meningoencephalitis in HIV/AIDS: when to start antiretroviral therapy?

**DOI:** 10.1186/s12941-017-0184-2

**Published:** 2017-03-06

**Authors:** Carlos Franco-Paredes, Daniel B. Chastain, Alfonso J. Rodriguez-Morales, Luis A. Marcos

**Affiliations:** 1 Annals of Clinical Microbiology and Antimicrobials, Phoebe Putney Memorial Hospital, Albany, GA Mexico; 2 Hospital Infantil de Mexico, Federico Gomez, Mexico City, Mexico; 3 University of Georgia College of Pharmacy, Albany, GA USA; 4 Annals of Clinical Microbiology and Antimicrobials, Public Health and Infectious Diseases Research Group, Faculty of Health Sciences, Universidad Tecnologica de Pereira, Pereira, Risaralda Colombia; 5 Division of Infectious Diseases, Department of Medicine, Stony Brook University, New York, NY USA; 6 Direction of Scientific Research, School of Medicine, Faculty of Health Sciences, Universidad Tecnológica de Pereira, Floor 3, Office 14-315, La Julita, Pereira, 660001 Risaralda Colombia

The institution of antiretroviral therapy (ART) in HIV-infected individuals with the goal of achieving virologic control and restoring immunity has led to substantial declines in AIDS-related complications, non-AIDS related morbidity, and improved survival [1]. In addition to the benefit provided at the individual level, it is also a crucial intervention to reduce HIV-transmission. Current treatment guidelines continue to emphasize early initiation of ART among individuals presenting at any stage of HIV-infection regardless of their CD4 cell count [1].

There is also an overall consensus that ART should be initiated within the first 2 weeks in individuals with advanced HIV-infection presenting with an AIDS-defining opportunistic infection with the possible exception of cryptococcal meningoencephalitis. However, the most recent update of the treatment guidelines of the International Antiviral Society-USA recommend considering ART within 2 weeks of diagnosis of cryptococcal meningoencephalitis in resource-rich settings where there is increased availability of optimal antifungal therapy (amphotericin B formulations and flucytosine); and means to monitor and aggressively treat increased intracranial pressure [1].

Worldwide, the highest burden of CNS cryptococcosis occurs in Sub-Saharan Africa and Southeast Asia, however, a substantial burden of disease occurs in high-income settings [2–4]. In the US, the Southeast has the highest rates of AIDS-associated hospitalization and mortality due to cryptococcosis [5]. Restoring immune function by the institution of ART is a crucial intervention in HIV-associated cryptococcal meningoencephalitis [2, 3]. We suggest that the timing of ART initiation should be individualized in every case considering host and fungal-related ones.

## The intracranial life of *Cryptococcus*

In patients with HIV/AIDS, there are two distinct clinical scenarios of central nervous system (CNS) cryptococcosis: (a) cryptococcal meningoencephalitis or parenchymal presentations in the setting of advanced immunosuppression; and (b) cryptococcal immune reconstitution inflammatory syndrome (IRIS) after the initiation of ART (Table 1).

**Table 1:** Differences in the pathogenesis and clinical manifestations of untreated CNS cryptococcosis and cryptococcal associated IRIS in patients with HIV/AIDS

Features	HIV/AIDS associated central nervous system Cryptococcosis	Cryptococcal immune reconstitution syndrome
Pathogenesis	*Cryptococci* crosses the microvascular endothelium of the blood brain barrier (BBB) of pial vessels and penetrating arterioles and capillaries via a transcellular pathway There is no disruption of the blood–brain-barrier Polysaccharide antigen and yeast accumulation in subarachnoid space affecting the reabsorption process of the CSF in arachnoid villi There is some evidence to suggest that the large number of yeasts residing in the perivascular spaces and brain parenchyma may affect the drainage of interstitial fluid into the perivascular spaces and therefore contributing to intracranial hypertension	Triggered by accumulation of cryptococcal polysaccharide in the subarachnoid space due to its decreased clearance producing rapid chemokine-mediated monocyte recruitment into the subarachnoid space leading to leptomeningitis This immunological response is dysregulated and causes inadequate cryptococcal killing and clearance of the fungus within the central nervous system
Clinical spectrum of disease	Meningoencephalitis with symptoms predominantly caused by increased intracranial hypertension (headache, nausea, decreased hearing, decreased vision, and others) and less frequently of meningitis (fever and meningismus) Parenchymal forms (cryptococcomas) with symptoms of increased intracranial pressure and mass effect (i.e., seizures, brain herniation syndromes) Cerebrospinal fluid analysis with a paucity of white cells Cerebrospinal fluid culture with growth of *Cryptococci*	Meningitis manifesting in individuals receiving antifungal therapy and sudden onset of clinical neurologic deterioration after initiation of antiretroviral therapy (paradoxical IRIS) Meningitis with increased intracranial pressure among individuals with HIV and already receiving ARTs (unmasked IRIS) Cerebrospinal fluid analysis with a more inflammatory pattern (increased white cells) Cerebrospinal fluid culture with no growth
Neuroimaging	Dilated Virchow Robin spaces in T2-weighted MRI imaging in basal ganglia and brain steam but in some cases also throughout cerebrum without evidence of leptomeningitis in most reported case series In parenchymal forms, the confluence of gelatinous pseudocysts may produce cryptococcomas	Leptomeningitis in MRI (T1-weighted images with contrast)
Management	Antifungal therapy (induction, consolidation, suppression) and evacuation of CSF to reduce intracranial hypertension	Continuation of antifungal therapy CSF evacuation if indicated Corticosteroids

### Cryptococcal meningoencephalitis in advanced HIV-infection

**Fig. 1:**
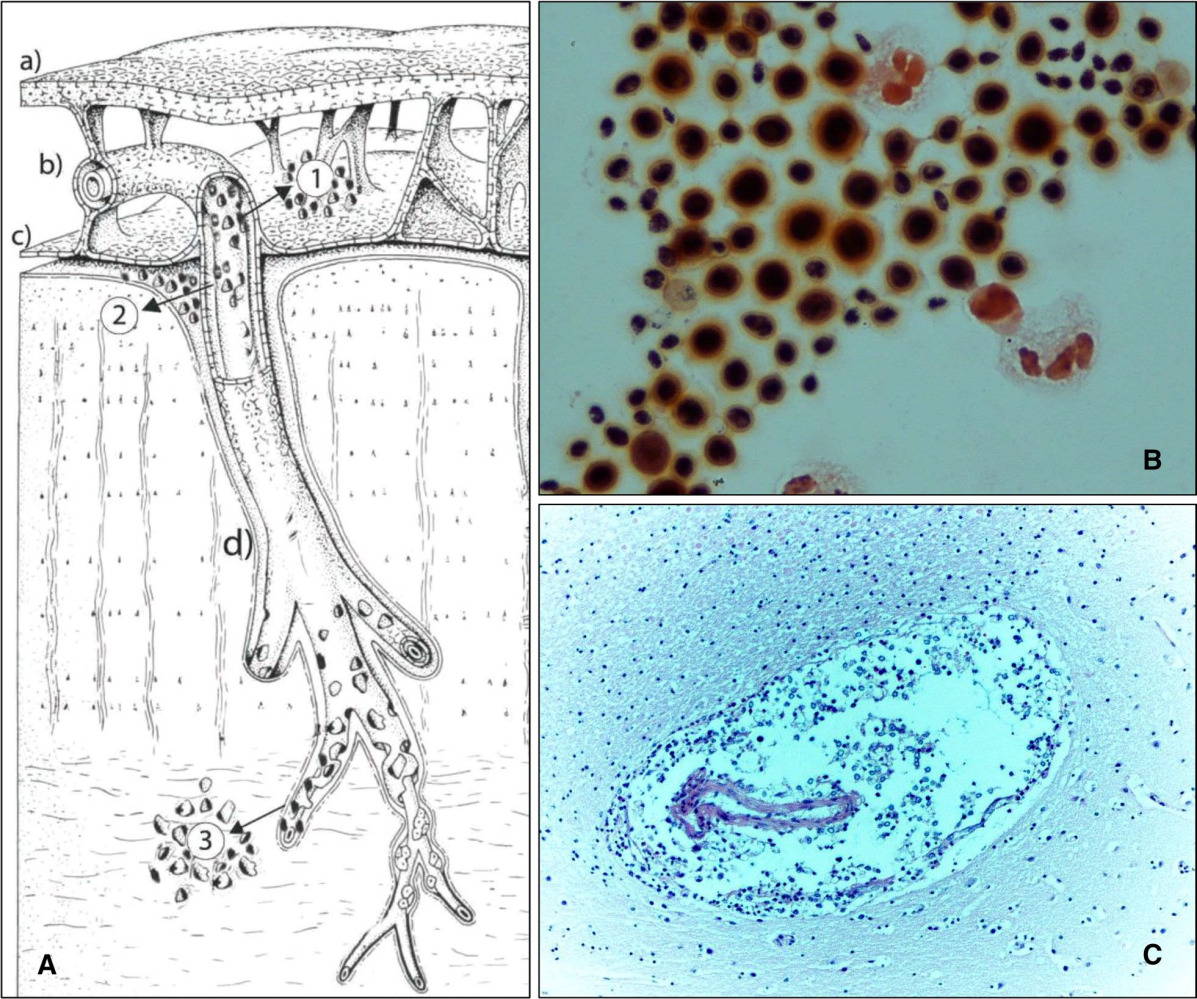
**A** Pattern of invasion of *Cryptococcus neoformans* into intracranial compartments in the setting of advanced HIV-infection*. *a* Arachnoid; *b* subarachnoid space; *c* pia mater; *d* brain parenchima; *1 Cryptococci* exiting arteries into the subarachnoid space; *2 Cryptococci* exiting pial arterioles into the perivascular spaces; and *3 Cryptococci* exiting parenchymal capillaries (*Modified and adapted from Reference [10]). **B** Gram-staining of cerebrospinal fluid (CSF) of a 32-year old female demonstrating large number of yeasts (oil immersion 10 × 100). She presented with a 6-week history of headache, episodes of confusion, and severe nausea. Her Cryptococcal antigen titer in CSF was >1:2200. She was found to be HIV-infected with a CD4 cell count of 2 cells/µL. **C** Micrographs of brain parenchyma demonstrating *Cryptococcus* identified in a perivascular space (H&E staining 40× magnification)

*Cryptococci* encounters limited immunological resistance during its route of entry into a human host with advanced HIV-associated immunosuppression. *Cryptococcus* establishes pulmonary infection through inhalation of its spores or desiccated yeast cells [5–7]. Once it reaches the lung parenchyma, *Cryptococci* enters the bloodstream and travels to the CNS [5–9]. The CNS vasculature system plays a crucial role in the mechanism of invasion of this fungal pathogen [9]. After large arteries from the carotid and vertebral circulations merge at the Circle of Willis, medium-size cerebral arteries branch into smaller pial arteries and arterioles that run along the surface of the brain. Pial arteries are constituted by an endothelial cell layer, a smooth muscle cell layer and an outer adventitial layer of leptomeningeal cells [10]. The adventitia is separated from brain tissue by the Virchow-Robin space as these arterioles penetrate deeper into the brain parenchyma (Fig. 1A). The Virchow-Robin space surround the walls of arteries, arterioles, veins, and venules as they course from the subarachnoid space and while penetrating through the brain parenchyma and plays an important role in the drainage of interstitial fluid from the brain parenchyma [10].

*Cryptococci* enters the cerebrospinal fluid (CSF), perivascular spaces, and brain parenchyma via transcellular crossing of the endothelial cells of blood brain barrier (BBB) but not of the blood-CSF barrier at the choroid plexus, and importantly, without affecting the integrity of the BBB [9, 11]. Other potential mechanism for entry into the CNS includes a “Trojan horse” whereby *Cryptococcus* enters hidden inside mononuclear cells [6]. Apparently, *Cryptococci* has avidity for the neurotransmitter-rich cerebral microenvironment surrounding the pial arterioles penetrating the brain parenchyma and for reaching the safe haven of the perivascular spaces [6, 12] (Fig. 1A). Indeed, leptomeningeal cells around arteries and arterioles in the human brain and in the subarachnoid space contain a high concentration of catechol-O-methyltransferase and pial arteries are densely innervated by perivascular nerves [10] providing a suitable environment where this yeast can procure substrates to synthetize melanin and overcome oxidative stress and phagocytosis [6].

In patients with HIV/AIDS, surgical neuropathological examinations and autopsy case series have consistently reported large numbers of yeasts in the subarachnoid space (Fig. 1B) and in the perivascular spaces (gelatinous pseudocysts or flask abscesses), particularly along the lenticulostriate arteries entering the basal ganglia through the anterior perforated substance [13–17]. Additionally, these reports have consistently demonstrated a paucity of inflammation in the meninges and subarachnoid space with only limited numbers of lymphocytes and few plasmocytes, and therefore the concept of “cryptococcal meningitis” may in fact constitute a misnomer [13, 14]. Since pia mater separates the subarachnoid space from underlying brain; and CSF from the interstitial fluid [18], *Cryptococci* likely enters different spaces at different segments of the microvasculature [10]: (a) during the trajectories of pial arterioles inside the subarachnoid space allowing access of the yeast into the CSF; (b) through penetrating arterioles after the subpial space allowing *Cryptococci* entering the perivascular spaces (Fig. 1C); and (c) via parenchymal capillaries that facilitates for this fungus, albeit in lesser degree, entering into the brain parenchyma (Fig. 1A) [10]. In support of the above pathways of invasion and pathological descriptions of CNS cryptococcosis, neuroimaging case series described dilated perivascular as punctate or oval hyper intense areas on T2-weighted images in the basal ganglia and brainstem. In some cases, high fungal burden promotes confluence of gelatinous pseudocysts and extend into the brain parenchyma leading to appearance of cryptococcomas [19–23]. The fungal burden identified in many patients may reach a degree of more than 1 million yeasts per milliliter of CSF, with greater CSF polysaccharide antigen titers, and higher degrees of intracranial pressures Abnormal dural or leptomeningeal enhancement is rarely described in these case series [8].

The spectrum of disease caused by CNS cryptococcosis in advanced HIV-infection is mostly due to intracranial hypertension caused by CSF outflow obstruction (i.e. subacute onset of headache, nausea, vomiting, fever, decreased hearing, decreased vision, seizures, or altered mentation) and less frequently symptoms of meningeal inflammation [12]. The precise mechanism for ICH is not fully elucidated, however, a reduced rate of removal of CSF precipitated by CSF outflow blockage caused by deposition of capsular polysaccharide and yeasts at arachnoid villi [12, 24–31]. In addition, drainage of interstitial fluid into the perivascular spaces is disrupted by the large number of yeast present in both the perivascular spaces and in the brain parenchyma [18, 32, 33] (Fig. 1A). Some individuals may manifest with parenchymal brain involvement with focal neurological signs caused by expanding cryptococcomas [19–23]. In summary, the spectrum of clinical, pathological, and imaging of CNS cryptococcosis in the setting of HIV/AIDS reflects the pathological mechanism of invasion of this fungal pathogen leading to a high fungal burden, a paucity of inflammation in the subarachnoid space and meninges, and substantial alterations in the dynamics of CSF and interstitial fluid homeostasis resulting in increased intracranial pressure [12].

### Cryptococcal immune reconstitution inflammatory syndrome

Despite its sophisticated role, the brain is vulnerable to events that produce rapid increases in intracranial pressure with potential devastating consequences [34]. This susceptibility resides in the fact that the brain is contained in a rigid and rudimentary cranium that restricts any increase in volume including brain edema or any alteration of the fine balance of the production, circulation or reabsorption of CSF [34, 35]. The expansion of one of the intracranial component (i.e., cerebrum, cerebrospinal fluid or intravascular blood) is at the expense of a reduction in another component. In this context, immune recovery associated to the initiation of ART in patients with cryptococcal meningoencephalitis may sometimes precipitates an exuberant cellular and molecular inflammatory battle against *Cryptococci* including components of its capsular polysaccharide [7, 8]. This response occurs predominantly in the subarachnoid space, leptomeninges, or in the Virchow-Robin spaces, which may disrupt the intracranial volume balance with potential life-threatening consequences.

With the increasing deployment and scale-up of ART in many settings, there is an overarching urgency to initiate ART in those identified with HIV-infection including those with advanced immunosuppression [1]. Patients with AIDS presenting with cryptococcal meningoencephalitis and CD4 cell counts <100 cell/µL, ART should be initiated as soon as possible. However, there are competing risks and benefits that must be balanced. In one hand, starting ART within the first 2 weeks of antifungal therapy may paradoxically induce restoration of pathogen specific immunity leading to cryptococcal IRIS which can be detrimental in a patient population that is immunologically unstable and clinically fragile [7, 8]. On the other hand, starting ART later may increase the chances of delaying fungal clearance and of the development of other life-threatening opportunistic infections. Cryptococcal associated IRIS occurs in approximately 13–30% of HIV-infected individuals and most cases occur in the first few months after initiating ART [2, 36]. Pathogen and host factors are important players in leading to IRIS: a high fungal burden and a poor proinflammatory response present prior to instituting ART (*Cryptococcus neoformans* promotes Th2 immune responses) [7, 8, 36]. In African cohorts, approximately one third of cases of CNS cryptococcosis present in patients already receiving ART. The institution of ART in this patient population may clinically unmask in the form of an IRIS, the previously undetected residence of *Cryptococci* in the CNS [7, 8]. Therefore, prior to starting ART, screening for asymptomatic cryptococcal antigenemia with lateral flow assays is considered best practice [8].

Cryptococcal associated IRIS is characterized by accumulation of cryptococcal polysaccharide in the subarachnoid space producing rapid chemokine-mediated monocyte recruitment into the subarachnoid space and CD4 cell redistribution into the CNS [2, 36]. This immunological response is dysregulated and causes inadequate cryptococcal killing and clearance of the fungus within the neuraxis. Patients with HIV/AIDS and with high fungal burden at the time of diagnosis seem to be at the highest risk of IRIS, particularly with rapid restoration of immune activity following ART [37, 39, 40]. However, most of the reports of IRIS associated with CNS cryptococcosis appear to be meningeal involvement in terms of its clinical presentation and also by evidence of leptomeningitis in T1-weighted contrast images [22]. CSF examination reveals important degrees of pleocytosis and high opening pressures but negative cultures [2, 36]. Cryptococcal associated IRIS may manifest with a constellation of symptoms associated with leptomeningitis and intracranial hypertension [38, 39]. This abrupt process occurring a few weeks or months after the institution of ART may manifest clinically with clinical neurologic deterioration; and sometimes with seizures, visual and hearing loss, or mass effect with herniation. CNS cryptococcosis associated IRIS should be considered in patients with HIV infection meeting all or some of the following criteria [38]: (a) CSF culture-confirmed first episode of cryptococcal meningoencephalitis, (b) resolution of cryptococcal meningoencephalitis symptoms before starting ART, (3) self-reported adherence to antifungal therapy and ART, (4) recurrence of symptoms (headache, nausea, vomiting, visual disturbance or others) after initiation of ART, (5) evidence of immunological and/or virologic response to ART, and (6) no alternative diagnosis found on laboratory testing and repeated clinical assessment. These clinical diagnostic criteria are imprecise but provide a framework for clinicians to consider this diagnostic possibility. Recent evidence suggests that starting ARTs within 1–2 weeks of diagnosis of CNS cryptococcosis is associated with excess mortality compared to starting ARTs at 5 weeks [8, 40]. The excess of deaths in the early ART group appeared to be caused by cryptococcal meningoencephalitis and not from Cryptococcal IRIS [8, 40]. Although the incidence of Cryptococcal associated IRIS was not different in the two groups, the findings of this study does suggest that ART instituted within 1–2 weeks does not have a meaningful impact in reducing CNS cryptococcosis associated mortality.

We suggest that the timing of initiation of ART should be individualized considering host factors such as the degree of inflammatory response (level of CSF pleocytosis and C-reactive protein level) and the fungal burden (cryptococcal antigen in CSF at the time of diagnosis and initial opening pressure); or assessing the sterilization of CSF at the end of the second week of optimal antifungal therapy. We believe that starting ART at 5 weeks or later should be considered particularly for those patients with: (a) extremely low CD4 cell counts; (b) cryptococcosis unveiling previously undiagnosed HIV infection; (c) CSF culture positive at 2 weeks despite standard antifungal treatment; and d) among those with high fungal burden as suggested by initial high opening pressure combined with cryptococcal antigen titer in CSF > 1:1056 [37].

Globally, AIDS-associated cryptococcal meningoencephalitis is a deadly disease causing approximately 20–25% of HIV-related mortality [2]. The initiation of fungicidal therapy with amphotericin B and flucytosine “stirs the pot” in the affected CNS areas (Fig. 1A) by promoting cell death and releasing large amounts of capsular polysaccharide. During the induction phase of antifungal therapy, efforts should focus on ensuring optimal medical management and ensuring sterilization of CSF while aggressively managing intracranial hypertension with CSF drainage. In this context, deferring ART for 5 weeks or longer, may offer a margin of safety to reduce the chances of IRIS occurring in the confined space of the cranium.

Finally, the persistent occurrence of cryptococcal infection in individuals with HIV-infection living in high-income settings unveils two major concerns: (a) the large number, in some settings, of undiagnosed cases of HIV infection who are unaware of their infection; and (b) the complex interplay of individual, social, and healthcare barriers facing individuals living with HIV-infection to enter into the HIV continuum of care.
